# Supplementary material for: “Does a Respiratory Virus Have an Ecological Niche, and If So, Can It Be Mapped?” Yes and Yes
Source: Trop Med Infect Dis. 2023 Mar 17;8(3):178. doi: 10.3390/tropicalmed8030178 (PMC10055886; doi:10.3390/tropicalmed8030178)
Supplement: Supplementary file 1 [file tropicalmed-08-00178-s001.zip › tropicalmed-2103204-supplementary.pdf]

Supplementary material

Time dependent models

Here we offer more detail as to how time dependence is included into an SDM/ENM. We consider a time dependent variable of interest, $v(t)$, defined by an epidemiological "who" ensemble, such as confirmed cases, deaths for age group > 60, mortality etc. The variable of interest may be ordinal, nominal or interval, and extensive or intensive. Its calculation requires a discretisation in time, so we may consider N_T time intervals, each of duration δt, to yield a total time period $\Delta_T = N_T * \delta t$. We may consider a variable $v(t, \delta t)$ specified over an interval of time δt, such as the number of confirmed cases of COVID-19 in the ith time interval, δt_i, or over the entire period, Δ_T. We may further consider $v_\alpha(t, \delta t)$, now calculated for a spatial cell α on a grid partition of N_s spatial cells on a region of interest. For instance, the number of confirmed cases in a country, α, in a month, δt, beginning at t being the first of June, 2021. Thus, $(\alpha, \delta t_i)$ defines a spatio-temporal cell, to which we can assign a value of $v(t)$, $v(\alpha, \delta t_i)$. A target class C can be formed via any criterion that specifies a subset $\mathcal{S}_C \times \mathcal{T}_C \subset \mathcal{S} \times \mathcal{T}$ of cells. For instance, those spatial cells that have confirmed cases in the time interval δt_i, or the 10% of spatial cells that have the highest mortality, as calculated in the interval δt_i. Taking the discretisation $(\alpha, \delta t_i)$ as the finest resolution, then $v(\Delta S, \Delta T)$ can be calculated for any spatial region ΔS and time interval ΔT. In the case of an extensive variable, such as number of deaths, this corresponds to a simple aggregation.

Turning now to the habitat variables, $\mathbf{X}$; in the same way, we can define a habitat variable $X_i(\alpha, \delta t_i)$ on a spatial cell in the time interval δt_i. It may be that $X_i(\alpha, \delta t_i) = X_i(\alpha)$, i.e., it is independent of time over the interval of interest. For instance, over the lifetime of the pandemic we may take a climatic variable, such as average annual temperature, to be constant. On the other hand, if $X_i(\alpha, \delta t_i)$ represents the number of confirmed cases in the interval δt_i, to be used as a niche variable, then certainly it will not be constant. The validity of an assumption of equilibrium depends on the rates of change and the magnitudes of change of C and $\mathbf{X}$ over the time period of interest. If the changes are slow and small over this period, then equilibrium may be a good approximation. Clearly, many variables of interest for COVID-19 are changing rapidly, such as vaccination rates, social distancing measures, travel restrictions etc.

Considering first a purely spatial model, we calculate $P(C(t)|\mathbf{X}(t))$ using the formalism of Method section. This will be calculated using data for a period Δ_T. A score for a niche variable $X_i^m(t)$ is given by $s(X_i^m(t)) = \ln(\frac{P(X_i^m(t)|C(t))}{P(X_i^m(t)|\bar{C}(t))})$, where $P(X_i^m(t)|C(t)) = N(X_i^m(t)C(t))/N(C(t))$ and $P(X_i^m(t)|\bar{C}(t)) = (N(X_i^m(t)) - N(X_i^m(t)C(t)))/(N - N(C(t))$. $N(X_i^m(t)C(t))$ is the number of cells with a co-occurrence of $X_i^m(t)$ and $C(t)$. For example, $C(t)$ could represent the 10% of cells (Mexican municipalities) with the highest number of cases in month t, while $X_i^m(t)$ could represent a dynamic habitat variable, such as the 10% of municipalities with the highest average mobility in that month, or a more static variable, such as the 10% of municipalities with the highest average income, or those municipalities that have pixels from an average annual temperature raster in the range $20.6 - 22.3 \deg C$. $N(X_i^m(t)C(t))$ thus counts, for example, the number of municipalities that are in the top 10% with the highest number of cases in month t and that also are in the top 10% of municipalities with the highest intra-municipal daily labor flows. We can calculate $s(X_i^m(t))$ in different time intervals, t_i, and compare $s(X_i^m(t_i))$ with $s(X_i^m(t_j))$. This can be done for all X_i^m and the degree of correlation in time calculated.

Although $X_i^m(t)$ and $C(t)$ may change in time, this does not imply that the ENM $P(C(t)|\mathbf{X}(t))$ itself changes in time. The difference between $s(X_i^m(t_i))$ and $s(X_i^m(t_j))$, however, will inform us of any changes in the ENM itself. In the case that the niche is conserved, i.e., $s(X_i^m(t_i)) \sim s(X_i^m(t_j))$, to predict the spatial distribution of the target class at t', we use the scores, $s(X_i^m(t))$, from the ENM at time t. For a given spatial cell, α at time $t' > t$, we associate the score contribution $s(X_i^m(\alpha, t'))$. Thus, for example, if X_i is divided into deciles, X_i^m, $m = [1, 10]$, a given spatial cell at time t may be associated with decile m, whereas at time t' it may be associated with a different decile m', so that the cell α has become more niche-like/anti-niche-like in the cases that $s(X_i^m) < s(X_i^{m'})$ or $> s(X_i^{m'})$ respectively.

Niche conservation implies that for a given habitat state, $\mathbf{X}$, then $P(C(t)|\mathbf{X})$ will not depend on time, i.e., there is the same probability to find the target species at any time t. When the niche is not conserved, however, then $P(C(t)|\mathbf{X}) \neq P(C(t')|\mathbf{X})$, i.e., the probability to find the target species given a certain habitat changes. In this case, the ENM $P(C(t)|\mathbf{X}(t))$ will have limited predictability for $t' > t$. To handle this situation we must only use $P(C(t)|\mathbf{X}(t))$ to predict into the future with a time horizon $\Delta_H = (t' - t)$ such that $s(X_i^m(t_i)) \sim s(X_i^m(t_j))$. We may then create a new model at $t + \Delta_H$, $P(C(t + \Delta_H)|\mathbf{X}(t + \Delta_H))$ and use it to predict at $t + 2\Delta_H$ and so on. In terms of a SDM, the fact that the habitat is changing means that spatial cells where the species was present/absent at t may now exhibit absence/presence. A useful way to quantify this, instead of using as target where the species is present in a given time period, is to focus on the *changes* between one period and the next. For example, we can take as target $\Delta_C(t, t')$ those spatial cells where the species was present/absent in period t but then is absent/present in period t'. An explicit example is to consider those spatial cells where there were no confirmed cases in period $t - 1$ but there were cases in period t. The data for the pair of periods $t - 1$ and t is taken as training data and the corresponding scores calculated. The target class, $\Delta_{C_{01}}(t, t - 1)$, now consists of those spatial cells where there was an absence, $C(t - 1) = 0$, in period $t - 1$ and a presence, $C(t) = 1$, in period t.

Thus, for a habitat variable X_i^m one calculates the score $s(X_i^m(t - 1)) = ln(P(X_i^m(t - 1)|\Delta_{C_{01}}(t, t -$

$1))/(P(X_i^m(t-1)|\Delta_{\bar{C}_{01}}(t,t-1))$, where $P(X_i^m(t-1)|\Delta_{C_{01}}(t,t-1)) = N(\Delta_{C_{01}}(t,t-1)X_i^m(t-1))/N(\Delta_{C_{01}}(t,t-1)$ and $P(X_i^m(t-1)|\Delta_{\bar{C}_{01}}(t,t-1)) = N(\Delta_{\bar{C}_{01}}(t,t-1)X_i^m(t-1))/N(\Delta_{\bar{C}_{01}}(t,t-1)$, where $\bar{C}_{01}(t,t-1)$ is the set complement of $\Delta_{C_{01}}(t,t-1)$. The scores $s(X_i^m(t-1))$ now define the ENM which can now be applied to the habitat variables $X_i^m(t)$ at time t to predict those cells that have an absence for confirmed cases at t but are predicted to have cases at $t+1$. Similarly, for the class variable being top 10% of municipalities with highest number of cases, we may consider $\Delta_{C_{01}}(t,t-1)$ to represent those municipalities that were not in the top 10% at $t-1$ but, due to a worsening epidemiological situation, passed into the top 10% at time t.

Finally, although we will not enter into detail here, we may also consider as habitat variables, changes in the habitat. For example, we may consider $\Delta_{X_i^{mm'}}(t,t')$, which represents those cells where there was a change in the habitat variable X_i from bin m' to bin m passing from period t' to period t.
